# The Role of Engineering Ethics in Mitigating Corruption in Infrastructure Systems Delivery

**DOI:** 10.1007/s11948-024-00494-0

**Published:** 2024-07-18

**Authors:** S. A. Ghahari, C. Queiroz, S. Labi, S. McNeil

**Affiliations:** 1 Lyles School of Civil Engineering, Purdue University, West Lafayette, IN 47907 USA; 2 World Bank, Washington, DC 20433 USA; 3 Department of Civil and Environmental Engineering, University of Delaware, 301 DuPont Hall, Newark, DE 19716 USA; 4 School of Civil and Environmental Engineering, University of New South Wales, Kensington, NSW, 2052, Australia

**Keywords:** Corruption, Ethics, Civil infrastructure, Values, Governance

## Abstract

Indications that corruption mitigation in infrastructure systems delivery can be effective are found in the literature. However, there is an untapped opportunity to further enhance the efficacy of existing corruption mitigation strategies by placing them explicitly within the larger context of engineering ethics, and relevant policy statements, guidelines, codes and manuals published by international organizations. An effective matching of these formal statements on ethics to infrastructure systems delivery facilitates the identification of potential corruption hotspots and thus help establish or strengthen institutional mechanisms that address corruption. This paper reviews professional codes of ethics, and relevant literature on corruption mitigation in the context of civil engineering infrastructure development, as a platform for building a structure that connects ethical tenets and the mitigation strategies. The paper assesses corruption mitigation strategies against the background of the fundamental canons of practice in civil engineering ethical codes. As such, the paper’s assessment is grounded in the civil engineer’s ethical responsibilities (to society, the profession, and peers) and principles (such as safety, health, welfare, respect, and honesty) that are common to professional codes of ethics in engineering practice. Addressing corruption in infrastructure development continues to be imperative for national economic and social development, and such exigency is underscored by the sheer scale of investments in infrastructure development in any country and the billions of dollars lost annually through corruption and fraud.

## Introduction

Civil infrastructure systems include transportation facilities for all modes, energy, water and wastewater plants and distribution networks, parks and public space, and public buildings. These are developed with the goal of satisfying some societal or economic needs (McCuen et al., 2011; ASCE, 2023). These goals can be traced back to overarching goals including human welfare and well-being, quality of life, and livability (Ghahari et al., 2023), which in turn, are rooted in basic human values—the primordial human need for shelter, food, water, safety and security, and respect, and dignity (Straub, 1964), as well as mobility. For this reason, the development and operation of civil infrastructure systems continue to enhance the lives of billions of people on the planet in a manner that has not only been longstanding (ASCE, 2008; Kirby, 1990; Petroski, 2001) but also is far reaching (ASCE, 2007; NAE, 2008).

Ironically, the development of civil infrastructure, for all its benefits to national economies and human quality of life, exposes society’s vulnerability to anti-social behaviors that impair the benefits of these systems, and in some cases, contravene the purpose for which they were built. These anti-social behaviors are largely characterized by illicit private gain at the expense of the public good thorough, for example, direct misallocation of public funds in a fraudulent, dishonest (and often, surreptitious) manner to individuals for payment for work done ostensibly to specification, but in fact falls outside the established acceptance criteria. Rose-Ackerman (1978) observed that corruption often occurs at the interface of the public and private sectors.

The economic and financial consequences of corruption can be as profound as its social, political, and environmental impacts. These occur through reduced economic development and reduced funding for investment or project implementation. It has been shown through research that higher levels of corruption are linked to lower investment and growth (Mauro, 1995; The World Bank, 1997), and favoring of inefficient producers, inequitable distribution of scarce public resources, and leakage of revenue from government hands to individual hands (Asian Development Bank, 2022). Transparency International (2020) stated unequivocally that corruption impairs economic development. Annually, more than $1 trillion (that is, 1.25% of global GDP) is lost due to collusion, corruption, and fraud (Mauro et al., 2019). Given that about 10% of the world population lives under the $2.15-a-day poverty line (World Bank, 2022), the magnitude of these losses is a stark reminder of the far-reaching adverse impacts of corruption in terms of the absence of social infrastructure that could otherwise benefit certain segments of the population (World Bank 2019). Corruption causes massive financial losses to governments that already struggle to deliver essential services to their citizens. Corruption also tends to threaten the financial stability of state-owned enterprises, as was the case of Petrobras, Brazil’s state-owned oil company. In one example, a high-level Petrobras executive was allegedly bribed about $4 million by Honeywell UOP (an oil company), to provide inside information and secret assistance, so Honeywell could win a contract, to the detriment of Petrobras (US Department of Justice, 2022; US Securities and Exchange Commission, 2022).

The objective of this paper is to discuss the problem of corruption in the context of engineering ethics and show how engineering ethics could be leveraged to help in the fight against corruption. The research approach starts by drawing from the literature, examples of mitigation strategies and then categorize these strategies in terms of their connection to ethical responsibilities, ethical principles, target actions, and relevant time frames. We do this to provide insights to help in implementation of mitigation initiatives. By setting the discussion of corruption in this context, we connect ethical principles and responsibilities, the propensity for corruption, and strategic, tactical, and operational mitigation actions. We then support these connections using in-practice examples from the literature. *Additional background and examples can be found in *Ghahari (2022)*.*

The paper is organized as follows: first, we present the broad background concepts for the study and the motivation for the paper including a definition of corruption and identification of its impacts. Next, we discuss values, value systems, and the properness triad (morality, ethics, and law). Then, we examine the connection between the principles of engineering ethics and corruption that subsequently leads into a review of the propensity for corruption. We then describe corruption mitigation initiatives and their connection to ethics and present examples of corruption mitigation initiatives in terms of ethical responsibilities, ethical principles, approaches, and scopes (strategic, tactical, or operational). In the last section, we offer some concluding remarks, and make some recommendations for the practice.

## Value Systems and the Properness Triad

### Value Systems

In corruption-related literature, researchers have typically connected corrupt behaviors to the paucity of ethics, morals, and values (Vee & Skitmore, 2003); Zou, 2006; Gundez & Onder, 2013; Owusu et al., 2019; Owusu et al., 2020). An explicit discussion of value systems and how these connect to the efficacy of corruption mitigation remains a clear lacuna in the literature. For this reason, we first identify opportunities to address corruption and then discuss the concept of value systems, and the so-called “properness triad” (that is, the triumvirate of morality, ethics, and law). In general, morals are interpreted as a sense of right or wrong. Ethics, on the other hand, is interpreted as the principles of “good” versus “malevolent” that are commonly agreed by public. A philosophical discussion on the morality and ethics is beyond the scope of this paper; however, we believe whatever agrees with moral is ethical and vice versa.

Arguably, any discussion of corruption needs to be set in the context of the broader overall values of the society in question. However, there is no question that certain behaviors are discouraged across different cultures and societies. Often, these core standards of behavior tend to stem from Mosaic law and other ancient texts and oral traditions including those that recognize supernatural deities. These behaviors, or “values”, have been passed on from ancient mores to modern-day societies. Over several millennia, societal values led to the development of customs, traditions, and laws within communities. Values can be defined as the set of preferences regarding what is appropriate and what is not in a society and represent an internal gauge for what is right or wrong. The types of values held by society include ethical, moral, religious, political, cultural, social, and aesthetic (McCuen et al., 2011). A community’s “value system” is a set of consistent values that are drawn from multiple types of values, and unlike personal values, tend to be generally stable across time or situations. With the formation of professions and organizations serving a common interest, values also led to the development of rules of behavior for members of that organization. Values that underlie the code of ethics of most professional societies include honesty, stewardship, responsibility, and discipline.

### The Properness Triad—Morality, Ethics, and Law

Human value systems, as they evolved over the millennia, seem to have been forged by a triumvirate of forces—morality, ethics, and law that existed in societies in any given era. Therefore, it can be argued the perpetrators of corrupt practices are fully conscious that their actions in this regard are immoral, unethical, illegal, or some combination thereof. Conversely, morality, ethics, and law are shaped by the extant societal values in any given era. Morality is what distinguishes between actions that are considered right by society and those that are wrong, and is often heavily influenced by religion and culture, particularly where explicit moral codes are established in religious texts or dictums to guide human behavior. The most famous example of a moral code is the Golden Rule: “Treat others how you wish to be treated.” Without a doubt, the perpetrators of corrupt actions would prefer to not be at the receiving end of corrupt actions to the extent that they were denied a due benefit or suffered an undue cost without their consent. Ethics is a branch of philosophy that addresses what can be considered right or wrong behavior. Law is a collection of rules that are enforced through social institutions to govern the behavior of individuals and organizations, and thus protects the individual or resources from the malicious actions of others. The importance of law in society is underscored by the fact that in most countries, the law-making body (or legislature) constitutes one of the three arms of government (the other two are the executive and the judiciary). As civil engineers plan, design, and operate civil systems, they routinely encounter situations related to morality, ethics, and law. For this reason, the issue of corruption in civil infrastructure development is not only a legal issue but one of morality and ethics. The morality-ethics-law triad could be viewed as a conceptual Venn diagram of three overlapping circles (each circle representing morality, legality, and ethicality), and each of the seven regions in the diagram representing one of several combinations associated with the legality, morality, or ethicality of an action.

## Infrastructure Development Phases and Their Vulnerability to Corruption

The development of civil infrastructure systems spans the phases of planning, design, construction, operations (including monitoring and maintenance), and end-of-life. Until the mid-twentieth century, most of these phases were carried out in-house by civil infrastructure agencies. However, gradually, the work execution at most phases has shifted to external (non-agency) entities through consulting or contracting arrangements. This trend is particularly the case for the construction phase. Of all the phases, the construction phase was the first to be executed by entities external of the agency. This was in circa 1765, when John Metcalf was contracted to construct 180 miles of turnpike road in England (Thomas, 1970). In the current era, major work at all development phases is carried out through such contracting arrangements. Most infrastructure agencies retain skeletal staff to oversee or supervise the work of the contractors and consultants at each phase, or to carry out routine work which in some cases are relatively minor and involve minimal capital outlay.

In this paper, we focus on the bidding (because it is a crucial preliminary stage), construction phase (because it is the most cost-intensive phase), and the operations and maintenance (O&M) phase (because it is the longest phase). Current practice is that work in both these latter phases is typically given out on contract: the external entity carries out the work while the agency monitors the work and retains ownership, so they work in tandem to ensure that a high level of service is provided to the infrastructure users. In some cases, the agency leases ownership to the contractor for a specified period, in addition to the O&M works and services. In a few cases, the work is done in-house. We discuss herein each of these delivery mechanisms.

### In-house Delivery of Construction, Operations, and Maintenance Works and Services

In-house delivery refers to the construction, operations, or maintenance of civil infrastructure systems where the agency uses its personnel, equipment, material, and other resources for the works. Currently, this delivery mechanism is often used for only relatively minor or routine maintenance work, and rarely used for construction. In-house project delivery is susceptible to corrupt behavior by the agency employees through over-invoicing of material purchases, pilfering, ghost workers, unauthorized sale of the site materials and equipment and pocketing the proceeds, and other misappropriation of resources intended for the project. It may very well be the case that such practices were what motivated the continued shift of civil infrastructure agencies towards contractual project delivery instead of in-house delivery of their physical activities.

### Contractual Delivery Through Public Private Partnerships (PPP) of Projects for Construction and Operation and Maintenance

Contractual project delivery is an approach by a civil infrastructure owner or operator for designing, constructing, operating, monitoring, or maintaining the infrastructure by entering into legal agreements with one or more parties. Depending on which phase(s) are included in the contract, the variants of contractual project delivery may include design-bid-build, design-build-operate-maintain, build-operate-transfer, build-own-operate-transfer, design-build-finance-maintain, design–build–finance–maintain-operate, and so on. This may be done in a traditional contracting context or under a PPP (or P3) arrangement (Li & Akintoye, 2003). In most PPP arrangements, the private sector typically provides upfront, the requisite capital financing for the government project and receives revenue (often through mutually agreed tolls or fees) from taxpayers and/or the infrastructure users over a given period (Caves, 2004).

Well-designed PPP arrangements can lead to benefits including (Mladenovic & Queiroz, 2014): (a) greater financial efficiency, by leveraging public money through the mobilization of private capital, reducing the impact of investments in infrastructure on the fiscal budget, and creating fiscal space to expand public service delivery in other sectors; (b) enhanced distribution of risks, by transferring design, construction, and performance risks to the private sector which is best able to manage such risks; and (c) improved governance, by increasing the accountability of the service provider through competitive bidding, disclosure policies, and public reporting. PPP arrangements may help overcoming the problems associated with not only in-house delivery but also other contracting approaches. However, PPPs come with their own devils. As evidenced in Brazil, renegotiations of infrastructure projects, particularly PPPs, open significant exposure to corruption. This was the cause of the largest corruption scandals in the history of the country. It was reported that the firm at the center of this scandal, Odebrecht, had obtained contracts through competitive processes but deliberately underbid the contract items. The contracts were subsequently renegotiated in favor of the firm. By Odebrecht’s admission in a U.S. District Court, the company paid about USD788 million in bribes in Brazil and 11 other countries, securing more than 100 contracts that generated USD3.3 billion in profit for the company (The World Bank, 2020a, 2020b).

## The Role of Engineering Ethics

### The Two Schools of Thought

In the field of moral philosophy, there exist two alternative normative theories of ethics regarding the basis for adjudging the rightness or wrongness of an action: deontological vs. consequential. Recognizing the complexities of ethical decision making, we briefly review these two theories. This review serves as a reminder that ethics involves balancing competing ideas and context specific issues but does not provide a detailed discussion of the theories and nuances.

Deontological ethics (synonyms: deontology, rule-based ethics) is that the morality of an action should be determined based on the rightness or wrongness of the action due to an established set of principles and rules, instead of the outcomes of the action. Ethical codes in civil engineering, like other professions, are driven by deontological ethics because they establish rules of behavior. From a *deontological perspective*, the *degree to which the ethical codes are followed* during the civil infrastructure development could serve as a general moral compass to adjudge whether any action or policy associated with the infrastructure development is good or bad.

Consequentialist ethics, on the other hand, assess the rightness or wrongness of an act based solely on the outcomes of the act. The development of any civil infrastructure system is driven by the agency’s (and other stakeholders’) goals and objectives which ultimately derive from the underlying values of society. These values serve as the basis on which civil engineers establish the performance outcomes of their infrastructure, and can be broadly classified as social, economic, and environmental, and may be beneficial or adverse. Therefore, from a *consequentialist perspective*, the *performance outcomes* of civil infrastructure development could individually or collectively serve as a general moral compass to adjudge whether any action or policy associated with the infrastructure development is good or bad.

It can be argued that the two normative theories, at least from the civil engineering perspective, are related: stronger deontological ethics can generally lead to superior consequentialist ethics. Ethical codes in civil engineering are driven by deontological ethics not consequentialist ethics. As such, in the next section, we expand the discussion of engineering ethics from the deontological viewpoint.

### Deontology as a Foundation for Engineering Ethics

As discussed in the previous section, from a deontological viewpoint, the goodness or badness of an action is determined based on the moral intentions behind the action. These intentions can be shaped by formal rules of behavior. However, as in most aspects of life, there exist grey areas that leave room for misinterpretation. In this respect, engineering ethical codes play a vital role because they specify an explicit list of dos-and-don’t-dos for the engineer with little room for misinterpretation. Professional engineering organizations in most countries have established codes of ethics by which their members regulate their work practices, conduct, and relationships. These organizations also urge engineers to hold themselves to the highest standards of professional and ethical behavior and recognize explicitly the obligation of individual engineers to uphold the integrity, self-respect, and honor of the engineering vocation by honest and impartial service to their employers, patrons, and the community.

Engineering ethics issues that arise in infrastructure development could be best addressed through the lens of engineering ethics explicitly established to connect specific actions in question, to the engineer’s responsibilities. These responsibilities, as implied in the ethical codes, include, for example, issuing public statements only in an objective and truthful manner, acting in professional matters for each client as faithful agents or trustees, and avoiding improper solicitation of professional assignments. Violations of these and other ethical principles have been identified as the direct or indirect underlying causes of corruption, including greed, and dishonesty (Owusu et al., 2019).

We discuss herewith some of these fundamental canons and rules of practice that are associated with civil engineering ethics, and how they are related to corruption. The discussion is built around the ASCE Code of Ethics but could also be applied to codes from other countries or professional organizations. These professional codes of conduct govern the obligations of registered engineers to society, their employers, and their clients and to other registered engineers, in that hierarchical order. These codes exhort members of the engineering profession to uphold high principles of integrity and honesty, that in providing their services, engineers must ensure fairness, impartiality, and equity, and always seek to safeguard public safety, health, and welfare. For example, in the United States, the preamble to NSPE’s Code of Ethics for Engineers (2019) states that “members of the profession recognize that their work has a direct and vital impact on the quality of life for all people.” Furthermore, any services rendered by engineers require “honesty, impartiality, fairness, and equity, and must be dedicated to the protection of public health, safety, and welfare” and “perform under a standard of professional behavior that requires adherence to the highest principles of ethical conduct.” In addition, in 2020, the American Society of Civil Engineers—(ASCE, 2020) released an updated code of ethics to reflect the technological changes by addressing “behavioral intent rather than prescriptive rules.” The preamble to the updated code states, “Members of the ASCE conduct themselves with integrity and professionalism, and above all else protect and advance the health, safety, and welfare of the public through the practice of civil engineering” and iterates the civil engineer’s responsibilities (in order of priority) to (a) society, (b) the natural and built environment, (c) the profession, (d) clients and employers, and (e) peers, as articulated in the code of ethics. Specific responsibilities speak directly to corruption (ASCE, 2020), for example: “zero tolerance for bribery, fraud, and corruption in all forms, and report violations to the proper authorities” (this reflect the engineer’s responsibility to society); “reject practices of unfair competition” (this reflect the engineer’s responsibility to the profession); “promote and exhibit inclusive, equitable, and ethical behavior in all engagements with colleagues” (this reflects the engineer’s responsibility to their peers); and “act with honesty and fairness on collaborative work efforts” (this also reflects the engineer’s responsibility to peers). Other examples of responsibilities related to the four fundamental principles, presented in Table 1, are related to corruption. Likewise, we believe every step towards having a less corrupt environment leads to sustainable development, either in the health sector (Mackey et al., 2018), energy sector (Lu et al., 2019), or civil engineering construction practices, as is discussed throughout this paper.

**Table 1:** Fundamental principles and examples of responsibilities in the ASCE Code of Ethics (ASCE, 2020)

Fundamental principle	Examples of responsibilities
Create safe, resilient, and sustainable infrastructure	First and foremost, protect the health, safety, and welfare of the public Enhance the quality of life for humanity Adhere to the principles of sustainable development
Treat all persons with respect, dignity, and fairness in a manner that fosters equitable participation without regard to personal identity	Treat all persons with respect, dignity, and fairness, and reject all forms of discrimination and harassment Recognize the diverse historical, social, and cultural needs of the community, and promote and exhibit inclusive, equitable, and ethical behavior in all engagements with colleagues
Consider the current and anticipated needs of society	Consider the capabilities, limitations, and implications of current and emerging technologies Consider and balance societal, environmental, and economic impacts, along with opportunities for improvement Mitigate adverse societal, environmental, and economic effects Use resources wisely while minimizing resource depletion Present clearly and promptly the consequences to clients and employers if their engineering judgment is overruled where health, safety, and welfare of the public may be endangered
Utilize knowledge and skills to enhance the quality of life for humanity	Uphold the honor, integrity, and dignity of the profession Promote mentorship and knowledge-sharing equitably with current and future engineers Educate the public on the role of civil engineering in society

Ethical codes require that engineers that are aware of infringements (or appearance thereof) related to any rules of professional conduct, report such violations to a specified authority. Such a responsibility is not a matter of personal choice but a professional obligation, as engineering professional societies recognize that the practice of professional engineering is not a right but a privilege. Also, these societies invariably charge engineers with the accountability of “adhering to the highest standards of ethical and moral conduct in all aspects of their professional practice” (Institution of Engineers, 2019), and in certain cases, their personal lives. As such, the privilege of engineering practice (often represented as a license to practice) could be withdrawn by the professional organization where the engineer’s behavior deemed unethical by the professional organization. In some countries such as Australia, there is explicit recognition in the code of ethics that engineers need to respect the dignity of the individual, and to act only based on a “well-informed conscience” (Institution of Engineers, 2019). The codes of ethics of NSPE, Institution of Engineers, and ASCE are generally similar to the civil engineering codes of other countries.

## Effectiveness of Ethics as a Corruption Mitigation Initiative

Corruption mitigation measures have been gaining momentum (Wolfensohn, 2020; Wang, 2020). In this section, we offer a classification of such measures: (a) policy statements, guidelines, and codes, (b) organizational and political structures, (c) monitoring and penalties, and (d) advanced technology. Monitoring and penalties, and advanced technology, which focus on enforcement and implementation, provide disincentives to corrupt practices at various phases of infrastructure life cycle. In this discussion, we focus on the first two classes of corruption mitigation, that is (a) and (b) above, providing examples, and connecting the examples to specific fundamental canons and rules of practice in ASCE’s Code of Ethics.

How effective are ethical codes in curbing corruption? This is an important question. In a quest for answers, researchers have used a variety of tools including literature reviews, surveys, focus-groups, workshops, and interviews. While the results from these previous studies may not be transferable to other contexts and locations, they suggest the opportunities to support other corruption mitigation measures and the role of ethical codes as a foundation for education and awareness.

Zou (2006) assessed the efficacy of corruption mitigation strategies and determined that the development of an honest and ethical culture in the construction industry, which we interpret to be founded on ethical codes, is imperative to support a long-term strategy. It has been argued that other ethics-based initiatives, including statements regarding corporate mission and values, customs, slogans, and role models, can be effective in establishing a moral tone within an organization to forestall corrupt practices (Luo, 2005)*.* Owusu et al. (2019) found that poor professional ethical standards continue to be the most cited cause of corruption and argued that ethics codes are a modestly effective anti-corruption measure.

Oladinrin and Ho (2016) discussed processes for responsibly implementing Codes of Ethics and proposed the European Foundation for Quality Management Excellence Model (EFQM model) as a suitable framework for assessing the extent to which codes of ethics are embedded in an organization and the organization’s performance in the context of ethics. Alkhatib (2017) and Alkhatib and Abdou (2018) established a framework for ethical judgment of behavior and actions that are typically carried out in the construction environment. The frameworks are based primarily on the ethical dimension of professional responsibility and provide the basis for judging actions as either ethical or unethical.

Overall, these strategic level corruption mitigation initiatives are consistent with the fundamental canons and rules of practice listed in the ethics codes of engineering organizations in several countries, including the United States (ASCE, 2020).

### Policy Statements, Guidelines, and Codes

International organizations have published policy statements, guidelines, codes, and manuals as part of their fight against corruption—see Table 2. In the construction industry, the implementation or embedding of ethics codes has received some attention (Ho & Oladinrin, 2019; Oladinrin & Ho, 2016). For example, in Hong Kong, construction companies that bid on government projects are required to demonstrate that they “possess codes of ethics so as to cultivate an ethical ethos both at the individual and company levels” (Ho & Oladinrin, 2019).

**Table 2:** Ethics-related Initiatives (Policy Statements, Guidelines, Codes and Manuals) Published by International Organizations

Initiative	Organization	Source
Recommendation for further combating bribery of foreign public officials	Organization for Economic Co-operation and Development (OECD)	OECD (2009)
“Zero tolerance” policy	World Economic Forum, Transparency International, and engineering and construction firms	WEF (2016)
Scrutiny of projects Sanctions system “Zero tolerance” policy	World Bank Group (WBG)	Integrity Vice Presidency, (2016); World Bank (2016)
Rules and recommendations to combat extortion and bribery	International Chamber of Commerce (ICC)	International Chamber of Commerce (2005, 2011, 2015)
Business principles for countering bribery	Transparency International	(Transparency International, 2010)
Global Compact	United Nations	Brun et al. (2011); UN Global Compact & Transparency International (2009)
Preventing and managing conflicts of interest in the public sector: good practices guide	World Bank, OECD and UNODC	World Bank (2020a, 2020b),
Partnering against corruption initiative (PACI)	World Economic Forum (WEF)	International Chamber of Commerce et al. (2008), WEF (2007, 2009)

### Organizational and Political Structures

The principles and processes inherent in democratic systems of governance have generally been associated with lower corruption (Kunicova & Rose-Ackerman, 2005; Montinola & Jackman, 2001). The reasons for this include partisan rivalry, scrutiny of public officials, demands for open decision making and access to information, and electoral procedures, all of which tend to discourage corrupt practices. Corruption could be significantly lowered by improving planning and budgeting processes and their transparency, raising awareness of civil society’s contribution and financial auditing agencies, and reducing the unrestricted power of individual bureaucrats and unnecessary regulation (Lederman et al., 2005; Svensson, 2005; Van Rijckeghem & Weder, 2001). For example, avoiding no-bid contracts and nepotistic awards of government contracts. Implementation of open procurement practices has been shown to reduce corruption and contract amendments (Forcada et al., 2017; Owusu et al., 2019). However, privatization of construction firms generally leads to more complexity and possibly low transparency in the bidding process and contracting (Copplestone, 2006). Governments that function as both regulator and owner are often incapable of separating the two roles. The Infrastructure Transparency Initiative is a multi-stakeholder effort that leverages earlier efforts (such as Integrity Pacts) and tools (such as Open Government Partnership and Open Contracting Partnership) to enhance public accountability based on working groups, disclosure, and social responsibility (Ghahari et al., 2023; The World Bank et al., 2020a, 2020b). The institutional and organizational structures provide frameworks in which ethical responsibilities related to fair competition, equity, honesty, and fairness can be practiced in ways consistent with the engineering code of ethics.

### Examples of Mitigation Initiatives

Table 3 presents examples of mitigation initiatives that support the relevant codes of ethics. For each example, the table identifies the related ethical responsibility (society, profession, and peers), ethical principles (as presented in Table 1), approaches (preventive, educational or punitive), and scope (strategic, tactical, and operational). This structure can be used to help target appropriate corruption mitigation measures. Grace et al. (2016), in conjunction with the World Bank Group, developed a fully-automated classification system for identifying and measuring the risk of fraud, corruption, and collusion in international development contracts. For instance, zero tolerance policies have been noted by many agencies, such as WEF (2016) and World Bank (2016), to be a viable strategy of corruption mitigation, which can be implemented by high-level executives and be followed by peers in the profession in a punitive measure. Another corruption mitigation tactic noted in Table 3 is ensuring transparency in the planning process (Bertot et al., 2010), which needs to be carried out by the society, e.g. government, as a preventive cause. Open contracting using standard procurement documents is also another corruption mitigation operational method (World Bank, 2020a, 2020b) to be implemented by high level policy makers and followed by peers in the profession. Oversight and reporting systems, as a corruption mitigation tactic, (Theunynck, 2002; Wong & Guggenheim, 2005) are a preventive and educational approach to the problem, which needs to be implemented at all levels from high level policy makers to peers in the profession. Using best practices for maintenance, operations and monitoring (Gorgulu et al., 2020) is one of the other operational tactics to mitigate corruption; this is a preventive and educational method which needs to be implemented by high level policy makers and mid-level executives. Finally, proper management of materials (Yeheyis et al., 2013) is a preventive operational method for corruption mitigation which requires to be implemented at a high level.

**Table 3:** Corruption mitigation—examples, ethical responsibilities and principles, approaches, and scopes

Example	Source	Ethical Responsibilities			Ethical Principles				Approach			Scope		
		Society	Profession	Peers	Create safe, resilient and sustainable infrastructure	Treat all persons with respect, dignity and fairness	Consider the current and anticipated needs of society	Utilize knowledge and skills to enhance quality of life	Preventive	Educational	Punitive	Strategic	Tactical	Operational
Zero tolerance policies	WEF (2016), World Bank (2016), Integrity Vice Presidency (2016)		X	X		X					X	X		
Ensure transparency in the planning process	Bertot et al. (2010)	X					X		X				X	
Open contracting using standard documents	World Bank, 2020a, 2020b	X	X	X		X			X					X
Oversight and reporting systems	Theunynck, 2002, Wong & Guggenheim, 2005	X	X	X				X	X	X			X	
Using best practices for maintenance, operations and monitoring	Gorgulu et al., 2020	X	X					X	X	X			X	X
Proper management of materials	Yeheyis et al., 2013	X			X				X					X

## Concluding Remarks and Recommendations

Addressing corruption in infrastructure development continues to be imperative for national economic and social development. This exigency is underscored by the sheer scale of investments in infrastructure development at any country and the billions of dollars lost annually through corruption and fraud. Recognizing the strong connection between corruption and ethics, the paper is motivated by the realization that it could be worthwhile to address the problem of corruption in the context of engineering ethics. Accordingly, the paper shows how engineering ethics could be leveraged to help in the fight against corruption.

Through a review of published work, the paper identified some of the main corruption issues and discussed corruption in the context of engineering ethics, as well as the morality-ethics-legal triad. By setting the discussion of corruption in this context, the narrative connected the fundamental canons and rules of practice in civil engineering ethical codes on one hand, to corruption propensity on the other hand, to serve as a platform for recommending strategic, tactical, and operational mitigation actions. The paper then supported these connections using practical examples from the literature.

In practice, ethics, ethical behavior and codes of ethics are just one element of an effective corruption mitigation strategy that involves all stakeholders. As corruption places pressure on individuals as well as institutions, implementation of strategies grounded in ethics needs to be supported by ethical organizational cultures, whistleblower protections, and education and training. Furthermore, to be effective these actions are required in concert with regulatory reforms, inspection, and enforcement. It would be idealistic to think that ethics can solve the problem, but it should be foundation for solutions.

In view of the discussions in the paper, we make the following recommendations:

- Infrastructure owners and development institutions are encouraged to match the ethical codes of their professional staff to the various phases of their infrastructure systems delivery. This will facilitate identification of potential corruption hotspots and establish or strengthen institutional mechanisms that address corruption.
- As such, the framework and outcomes of this research can be used to guide a comprehensive assessment of opportunities to mitigate corruption through the connections to engineering ethics. The structure can be generalized to other mitigation strategies not explicitly discussed in this paper.
- The nuances of implementation will vary across the agencies, depending on their organizational structure, location, characteristics such as scale and timeline for the infrastructure system, and the willingness to educate the actors and enforce the principles.
- The paper’s framework focuses on engineering Codes of Ethics, but the principles are broadly applicable to other professionals, such as architects, public managers, financiers, and urban planners, involved in infrastructure systems delivery.
- Future research can be dedicated to carefully examine to what extent the ethics mechanisms in mitigating corruption presented in this paper can be effective. The effectiveness measures can be done by applying the mechanisms in real world projects, observing the outcomes, and getting feedback from the executives.

Acknowledging that there is no single solution to corruption, strategies based on ethics are just one of many approaches, and our recommendations related to ethics are somewhat obvious, this paper connects the concepts in terms of the importance of an ethical foundation, the phases of development infrastructure systems, and the relevant stakeholders.

## References

[CR1] Alkhatib, O. J. (2017). A moral (normative) framework for the judgment of actions and decisions in the construction industry and engineering: Part II. *Science & Engineering Ethics,**23*(6), 1617–1641. 10.1007/s11948-016-9851-5 27913987 10.1007/s11948-016-9851-5

[CR2] Alkhatib, O. J., & Abdou, A. (2018). An ethical (descriptive) framework for judgment of actions and decisions in the construction industry and engineering—Part I. *Science & Engineering Ethics,**24*(2), 585–606. 10.1007/s11948-017-9895-1 28321688 10.1007/s11948-017-9895-1

[CR3] ASCE. (2020). *Code of Ethics*, (pp. 1–2). American Society of Civil Engineers. https://www.asce.org/career-growth/ethics/code-of-ethics

[CR4] ASCE. (2023). About ASCE. https://www.asce.org/about-asce

[CR5] ASCE. (2007). *Vision for Civil Engineering in 2025*. Am. Soc. Civil Eng.

[CR6] ASCE. (2008). *Civil Engineering Body of Knowledge for the 21st Century* (2nd ed.). Am. Soc. Civil Eng.

[CR63] Asian Development Bank (2022). *Key indicators for Asia and The Pacific*, 53rd Edition. 10.22617/FLS220346-3. Retrieved from: https://www.adb.org/sites/default/files/publication/812946/ki2022.pdf

[CR7] Bertot, J. C., Jaeger, P. T., & Grimes, J. M. (2010). Using ICTs to create a culture of transparency: E-government and social media as openness and anti-corruption tools for societies. *Government Information Quarterly,**27*(3), 264–271. 10.1016/j.giq.2010.03.001 10.1016/j.giq.2010.03.001

[CR8] Brun, J.-P., Gray, L., Scott, C., & Stephenson, K. (2011). *Asset recovery handbook: A guide for practitioners*. World Bank Report. 10.1596/978-0-8213-8634-7

[CR67] Caves, R. W. (2004). *Encyclopedia of the city.* Routledge

[CR9] Copplestone, P. (2006). *Vietnam: The vital construction industry*. World Bank Report. 10.1016/j.giq.2010.03.001

[CR10] Forcada, N., Alvarez, A. P., Love, P. E., & Edwards, D. J. (2017). Rework in urban renewal projects in Colombia. *Journal of Infrastructure Systems,**23*(2), 04016034. 10.1061/(ASCE)IS.1943-555X.0000332 10.1061/(ASCE)IS.1943-555X.0000332

[CR11] Ghahari, S.A. (2022). *Detecting and measuring corruption and inefficiency in infrastructure projects using machine learning and data analytics*. Purdue University Graduate School. Thesis. 10.25394/PGS.15060534.v1

[CR12] Ghahari, S. A., Queiroz, C., Labi, S., & McNeil, S. (2023). Corruption propensity and mitigation at different phases of infrastructure development. *Journal of Public Works Management & Policy*. 10.1177/1087724X231162544 10.1177/1087724X231162544

[CR13] Gorgulu, N., Sharafutdinova, G., & Steinbuks, J. (2020). *Political dividends of digital participatory governance: Evidence from Moscow pothole management*. World Bank Report.

[CR14] Grace, E., Rai, A., Redmiles, E., & Ghani, R. (2016). Detecting fraud, corruption, and collusion in international development contracts: The design of a proof-of-concept automated system. In *2016 IEEE International Conference on Big Data (Big Data)*. 10.1109/BigData.2016.7840752

[CR15] Gunduz, M., & Önder, O. (2013). Corruption and internal fraud in the Turkish construction industry. *Science & Engineering Ethics,**19*(2), 505–528. 10.1007/s11948-012-9356-9 22371033 10.1007/s11948-012-9356-9

[CR16] Ho, C. M. F., & Oladinrin, O. T. (2019). A paradigm shift in the implementation of ethics codes in construction organizations in Hong Kong: Towards an ethical behavior. *Science & Engineering Ethics,**25*(2), 559–581. 10.1007/s11948-018-0026-4 29383559 10.1007/s11948-018-0026-4

[CR17] Integrity Vice Presidency, The World Bank. (2016). Annual Update Integrity Vice Presidency (Int). http://pubdocs.worldbank.org/en/118471475857477799/INT-FY16-Annual-Update-web.pdf

[CR18] International Chamber of Commerce. (2005). Combating extortion and bribery: ICCc rules of conduct and recommendations. https://iccwbo.org/content/uploads/sites/3/2005/10/Combating-Extortion-and-Bribery-ICC-Rules-of-Conduct-and-Recommendations.pdf

[CR19] International Chamber of Commerce. (2011). Corporate responsibility and anti-corruption: ICC rules on combating corruption. https://cdn.iccwbo.org/content/uploads/sites/3/2011/10/ICC-Rules-on-Combating-Corruption-2011.pdf

[CR20] International Chamber of Commerce. (2015). International Chamber of Commerce—Overview, functions, activities. https://corporatefinanceinstitute.com/resources/knowledge/other/international-chamber-of-commerce-icc/

[CR21] International Chamber of Commerce, Transparency International, The United Nations Global Compact, & The World Economic Forum Partnering Against Corruption Initiative (PACI). (2008). Clean business is good business: The business case against corruption. https://d306pr3pise04h.cloudfront.net/docs/issues_doc%2FAntiCorruption%2Fclean_business_is_good_business.pdf

[CR68] Institution of Engineers. (2019). *Code of ethics and guidelines on professional conduct* (pp. 1- 5). Engineers Australia.

[CR22] Kirby, R. S. (1990). *Engineering in history*. Dover.

[CR23] Kunicova, J., & Rose-Ackerman, S. (2005). Electoral rules and constitutional structures as constraints on corruption. *British Journal of Political Science,**35*(4), 573–606. 10.1017/S0007123405000311 10.1017/S0007123405000311

[CR24] Lederman, D., Loayza, N. V., & Soares, R. R. (2005). Accountability and corruption: Political institutions matter. *Journal of Economics & Politics,**17*(1), 1–35. 10.1111/j.1468-0343.2005.00145.x

[CR25] Li, B., & Akintoye, A. (2003). An overview of public-private partnership. In A. Akintoye, M. Beck, & C. H. Castle (Eds.), *Public-private partnerships: managing risks and opportunities. *Blackwell Publishing.

[CR26] Lu, J., Ren, C., Qiao, J., Yao, S., Strielkowski, W., & Streimikis, J. (2019). Corporate social responsibility and corruption: Implications for the sustainable energy sector. *Journal of Sustainability,**11*(15), 4128. 10.3390/su11154128 10.3390/su11154128

[CR27] Luo, Y. (2005). An organizational perspective of corruption. *Management and Organization Review,**1*(1), 119–154. 10.1111/j.1740-8784.2004.00006.x 10.1111/j.1740-8784.2004.00006.x

[CR28] Mackey, T. K., Vian, T., & Kohler, J. (2018). The sustainable development goals as a framework to combat health-sector corruption. *Bulletin of the World Health Organization,**96*(9), 634–643. 10.2471/BLT.18.209502 30262945 10.2471/BLT.18.209502 PMC6154071

[CR29] Mauro, P. (1995). Corruption and growth. *The Quarterly Journal of Economics,**110*(3), 681–712. 10.2307/2946696 10.2307/2946696

[CR30] Mauro, P., Medas, P., & Fournier, M. (2019). *The cost of corruption*. The International Monetary Fund.

[CR31] McCuen, R. H., Ezzell, E. Z., & Wong, M. K. (2011). *Fundamentals of civil engineering: An introduction to the ASCE body of knowledge*. CRC Press.

[CR32] Mladenovic, G. & Queiroz, C. (2014). Assessing the financial feasibility of availability payment PPP Projects. In *Proceedings of the 2*^*nd*^* T&DI Congress*, (pp. 602–611). 10.1061/9780784413586.058

[CR33] Montinola, G. R., & Jackman, R. W. (2001). Sources of corruption: A cross-country study. *British Journal of Political Science,**32*(1), 147–170. 10.1017/S0007123402000066 10.1017/S0007123402000066

[CR34] NAE. (2008). *The engineer of 2020: Visions of engineering in the new century*. National Academies Press.

[CR35] NSPE. (2019). *Code of ethics for engineers* (pp. 1–2). National Society of Professional Engineers.

[CR36] OECD. (2009). Recommendation of the council for further combating bribery of foreign public officials in international business transactions. https://www.oecd.org/corruption/anti-bribery/OECD-Anti-Bribery-Recommendation-ENG.pdf

[CR37] Oladinrin, O. T., & Ho, C. M. F. (2016). Enabling ethical code embeddedness in construction organizations: A review of process assessment approach. *Science & Engineering Ethics,**22*(4), 1193–1215. 10.1007/s11948-015-9679-4 26142742 10.1007/s11948-015-9679-4

[CR38] Owusu, E. K., Chan, A. P., Ameyaw, E. E., & Robert, O. K. (2020). Evaluating the effectiveness of strategies for extirpating corrupt practices in infrastructure project procurement. *Journal of Infrastructure Systems,**26*(2), 04020004. 10.1061/(ASCE)IS.1943-555X.0000531 10.1061/(ASCE)IS.1943-555X.0000531

[CR39] Owusu, E. K., Chan, A. P., & Darko, A. (2019). Thematic overview of corruption in infrastructure procurement process. *Journal of Infrastructure Systems,**25*(2), 02519001. 10.1061/(ASCE)IS.1943-555X.0000484 10.1061/(ASCE)IS.1943-555X.0000484

[CR40] Petroski, H. (2001). *The importance of engineering history*. International Engineering History and Heritage.

[CR41] Rose-Ackerman, S. (1978). *Corruption: A study in political economy*. Academic Press.

[CR42] Svensson, J. (2005). Eight questions about corruption. *The Journal of Economic Perspectives,**19*(3), 19–42. 10.1257/089533005774357860 10.1257/089533005774357860

[CR43] Straub, H. (1964). *A history of civil engineering—An outline from ancient to modern times*. MIT Press.

[CR44] Theunynck, S. (2002). *School construction in developing countries: What do we know* (pp. 1–37). World Bank Report.

[CR45] Thomas, R. J. S. (1970). Main Roads. *Journal of the Department of Main Roads, Sydney, New South Wales, Australia,**35*(4), 86–88.

[CR46] Transparency International. (2010). The 2010 U.K. bribery act adequate procedures: Guidance on good practice procedures for corporate anti-bribery programmes. https://www.transparency.org.uk/sites/default/files/Adequate_Procedures_Annexes_1-3.pdf

[CR69] Transparency International. (2020). *What is corruption?* Transparency International, Berlin, Germany. https://www.transparency.org/en/what-is-corruption#

[CR47] UN Global Compact & Transparency International. (2009). Reporting Guidance on the 10th Principle against Corruption. https://www.unglobalcompact.org/library/154.

[CR65] US Department of Justice (2022). Office of Public Affairs. *Honeywell UOP to pay over $160 million to resolve foreign bribery investigations in U.S. and Brazil*. Retrieved from: https://www.justice.gov/opa/pr/honeywell-uop-pay-over-160-million-resolve-foreign-bribery-investigations-us-and-brazil

[CR66] US Securities and Exchange Commission (2022). *SEC charges Honeywell with bribery schemes in Algeria and Brazil.* Press Release. Retrieved from: https://www.sec.gov/newsroom/press-releases/2022-230.

[CR48] Van Rijckeghem, C., & Weder, B. (2001). Bureaucratic corruption and the rate of temptation: do wages in the civil service affect corruption, and by how much? *Journal of Development Economics,**65*(2), 307–331. 10.1016/S0304-3878(01)00139-0

[CR49] Vee, C., & Skitmore, C. (2003). Professional ethics in the construction industry. *Engineering, Construction and Architectural Management,**10*(2), 117–127. 10.1108/09699980310466596 10.1108/09699980310466596

[CR70] Wang, J. (2020). *The global crisis of corruption.* Retrieved from https://gfintegrity.org/globalcrisis-of-corruption/

[CR50] WEF. (2007). Anti-corruption handbook: implementing the paci principles for countering bribery a manager’s guide for developing anti-corruption programs. https://www.compliance-instituut.nl/wp-content/uploads/PACI-Implementation-Handbook.pdf

[CR51] WEF. (2009). A Partner in Shaping History: The First 40 Years (1971–2010). World Economic Forum, https://www3.weforum.org/docs/WEF_A_Partner_in_Shaping_History.pdf

[CR52] WEF. (2016). Partnering against corruption initiative global principles for countering corruption: Application & general terms of partnership. http://www3.weforum.org/docs/WEF_PACI_Global_Principles_for_Countering_Corruption.pdf

[CR53] Wolfensohn, J.D. (2020). Combatting the cancer of corruption. https://www.worldbank.org/en/about/archives/history/past-presidents/james-david-wolfensohn

[CR54] Wong, S., & Guggenheim, S. (2005). Community-driven development: decentralization’s accountability challenge. In *East Asia decentralizes: Making local government work*, (pp. 253–267). http://siteresources.worldbank.org/INTEAPDECEN/Resources/Chapter-12.pdf

[CR55] World Bank. (1997). *World development report: The state in a changing world*. The World Bank Report.

[CR56] World Bank. (2016). The World Bank Annual Report 2016. http://pubdocs.worldbank.org/en/596391540568499043/worldbankannualreport2016.pdf

[CR57] World Bank. (2019). *Anticorruption Initiatives: Reaffirming Commitment to a Development Priority*. The World Bank.

[CR58] World Bank. (2020). Enhancing Government Effectiveness and Transparency: The Fight against Corruption. Global Report, September 2020. International Bank for Reconstruction and Development/The World Bank. https://www.worldbank.org/en/topic/governance/publication/enhancing-government-effectiveness-and-transparency-the-fight-against-corruption

[CR59] World Bank, OECD, & UNODC. (2020). Preventing and Managing Conflicts of Interest in the Public Sector: Good Practices Guide. https://documents.worldbank.org/en/publication/documents-reports/documentdetail/950091599837673013/preventing-and-managing-conflicts-of-interest-in-the-public-sector-good-practices-guide?deliveryName=DM85793

[CR64] World Bank (2022). *Fact sheet: An adjustment to global poverty lines*. Retrieved from: https://www.worldbank.org/en/news/factsheet/2022/05/02/fact-sheet-an-adjustment-to-global-poverty-lines.

[CR61] Yeheyis, M., Hewage, K., Alam, M. S., Eskicioglu, C., & Sadiq, R. (2013). An overview of construction and demolition waste management in Canada: A lifecycle analysis approach to sustainability. *Clean Technologies and Environmental Policy,**15*(1), 81–91. 10.1007/s10098-012-0481-6 10.1007/s10098-012-0481-6

[CR62] Zou, P. X. (2006). Strategies for minimizing corruption in the construction industry in China. *Journal of Construction in Developing Countries,**11*(2), 15–29.

